# The malaria parasite egress protease SUB1 is a calcium-dependent redox switch subtilisin

**DOI:** 10.1038/ncomms4726

**Published:** 2014-05-02

**Authors:** Chrislaine Withers-Martinez, Malcolm Strath, Fiona Hackett, Lesley F. Haire, Steven A. Howell, Philip A. Walker, Christodoulou Evangelos, Guy G. Dodson, Michael J. Blackman

**Affiliations:** 1 Division of Parasitology, MRC National Institute for Medical Research, Mill Hill, London NW7 1AA, UK; 2 Division of Molecular Structure, MRC National Institute for Medical Research, Mill Hill, London NW7 1AA, UK

## Abstract

Malaria is caused by a protozoan parasite that replicates within an intraerythrocytic parasitophorous vacuole. Release (egress) of malaria merozoites from the host erythrocyte is a highly regulated and calcium-dependent event that is critical for disease progression. Minutes before egress, an essential parasite serine protease called SUB1 is discharged into the parasitophorous vacuole, where it proteolytically processes a subset of parasite proteins that play indispensable roles in egress and invasion. Here we report the first crystallographic structure of *Plasmodium falciparum* SUB1 at 2.25 Å, in complex with its cognate prodomain. The structure highlights the basis of the calcium dependence of SUB1, as well as its unusual requirement for interactions with substrate residues on both prime and non-prime sides of the scissile bond. Importantly, the structure also reveals the presence of a solvent-exposed redox-sensitive disulphide bridge, unique among the subtilisin family, that likely acts as a regulator of protease activity in the parasite.

## Abstract (web summary)

In the malarial parasitophorous vacuole, the serine protease SUB1 processes parasite proteins that are required for release from host cells and invasion. Here, the authors report the first crystallographic structure of SUB1 in complex with its cognate prodomain revealing its substrate interactions and providing insight into its regulation.

## Graphical abstract

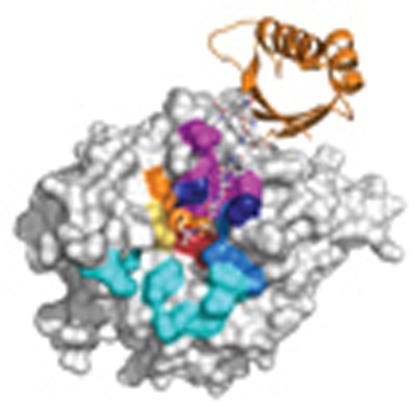

Around half the world’s population are at risk of infection with protozoan parasites of the genus *Plasmodium*, the causative agent of malaria. Blood-stage forms of the parasite invade erythrocytes, where they replicate within an intracellular membrane-bound compartment called a parasitophorous vacuole (PV). Each infected erythrocyte then ruptures, allowing egress of daughter merozoites which immediately invade fresh erythrocytes. Repeated cycles of intraerythrocytic replication and egress lead to a gradually increasing parasitaemia and clinical disease. This varies in severity depending on the *Plasmodium* species, but in the most virulent form of malaria, caused by *Plasmodium falciparum*, initial febrile episodes can quickly lead to severe pathology including anaemia, hypoglycaemia, respiratory distress, coma and other complications that are often fatal^1^.

Merozoite egress is a rapid, parasite-driven and temporally highly regulated process that involves a number of parasite and host-derived molecules^2^. Minutes before egress, a calcium- and cGMP-dependent signal triggers the discharge of a subtilisin-like serine protease called SUB1 from specialized secretory organelles of the still intracellular merozoite, called exonemes^3^,^4^,^5^. On its release into the PV lumen, SUB1 specifically cleaves a number of abundant merozoite surface and PV-resident soluble proteins involved in egress and invasion^3^,^4^,^5^,^6^,^7^. Pharmacological inhibition of SUB1 discharge or catalytic activity blocks egress and/or reduces the invasive capacity of released merozoites^3^,^6^. Drugs that target SUB1 activity should prevent disease progression and so have potential as a new class of antimalarial therapeutics, urgently needed in response to increasing drug resistance of the parasite^1^.

Orthologs of SUB1 are found in every *Plasmodium* species examined, suggesting a conserved role in egress. Features of the SUB1 primary sequence identify it as a member of the subfamily S8A of predominantly bacterial subtilisins^8^,^9^,^10^. Mapping of *P. falciparum* SUB1 (PfSUB1) cleavage sites in several of its endogenous substrates has enabled assembly of a consensus PfSUB1 recognition motif of Ile/Leu/Val/Thr-Xaa-Gly/Ala-Paa(not Leu)↓Xaa (where Xaa is any amino-acid residue and Paa tends to be a polar residue)^10^. An additional feature of the cleavage sites is the invariable presence of acidic (Glu, Asp) or hydroxyl-containing (Ser, Thr) residues at one or more of the proximal 5′-side positions flanking the scissile bond^8^. This observation, together with mutational studies and analysis of synthetic substrates based on these cleavage motifs, has indicated that—unusually for a S8A family subtilisin—substrate recognition by PfSUB1 requires interactions with both prime and non-prime-side residues of the cleavage site. An atomic resolution image of the architecture of the PfSUB1 active site is important for a full understanding of this unusual substrate specificity, and essential for the development of substrate-based inhibitors.

Like most subtilisins, PfSUB1 is synthesized as a zymogen, which undergoes maturation via autocatalytic cleavage of an inhibitory N-terminal prodomain^11^,^12^. Removal of the PfSUB1 prodomain occurs in the parasite endoplasmic reticulum^11^. The enzyme is then trafficked to the exonemes where it is stored until required at egress. The proteolytic activity of the mature enzyme is likely suppressed during storage to prevent autolysis, but it is unknown how this is achieved, or how the protease is activated on release into the PV just before egress.

Autocatalytic cleavage of the PfSUB1 prodomain is recapitulated in a baculovirus expression system, allowing production of correctly processed recombinant protein (rPfSUB1_p54_)^12^,^13^, which is largely still bound to its prodomain so is poorly enzymatically active. Earlier attempts to solve the structure of this protein were hampered by its heterogeneity and low solubility^13^. Recently, we found that limited proteolysis with chymotrypsin converts rPfSUB1_p54_ to a soluble, enzymatically active core catalytic domain of 38 kDa (called rPfSUB1_cat_) still partially bound to a 9-kDa prodomain fragment (Prod_p9_) but with no modification of its substrate specificity^10^. This material formed poorly diffracting crystals, the quality of which was improved by the addition of Fab fragments of the PfSUB1-specific monoclonal antibody NIMP.M7^4^. Here we describe the crystal structure at 2.25 Å resolution of the rPfSUB1_cat_–Prod_p9_–Fab complex. The structure explains how the unusual architecture of the PfSUB1 active site enables the interaction with sequences on both the non-prime and prime sides of the scissile bond. Remarkably, the structure also reveals a previously unsuspected labile disulphide bridge in the vicinity of the active site that regulates PfSUB1 enzymatic activity and may be of importance for enhancing enzyme activity on discharge into the PV.

## Results

### Overall rPfSUB1_cat_–Prod_p9_ fold

**Figure 1:**
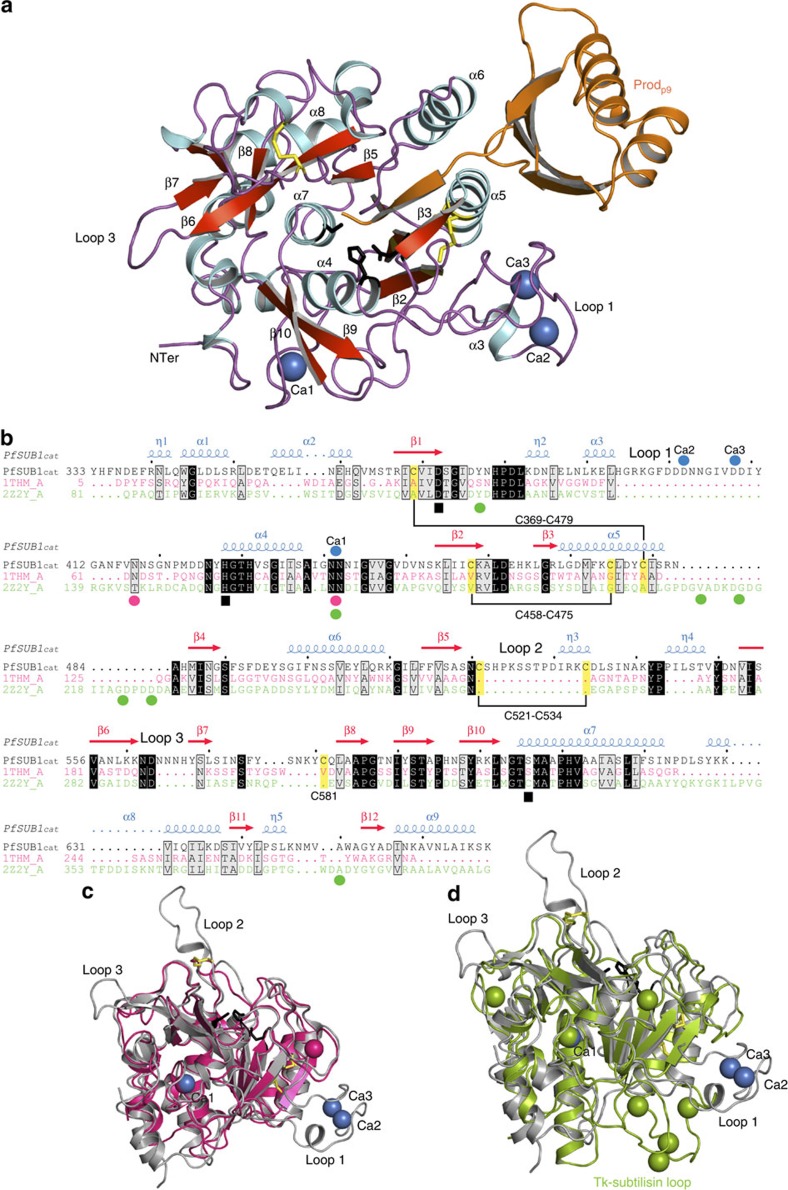
Structure of the rPfSUB1_cat_–Prod_p9_ complex. (**a**) Cartoon view of the crystal structure at 2.25 Å of the rPfSUB1_cat_–Prod_p9_–Fab complex (the Fab is not shown for clarity; see Fig. 2 for the complete structure). rPfSUB1_cat_ is coloured according to its secondary structure with numbered β-strands in red, α-helices in pale blue and loops in purple. Prod_p9_ is in orange, and the three bound Ca^2+^ ions are seen as blue spheres. The catalytic triad residues (Asp372, His428 and Ser606) are shown as black sticks and the three disulphide bridges are in yellow. (**b**) Primary sequence alignment of PfSUB1_cat_ with subtilisin S8B thermitase (PDB ID: 1THM, magenta, 29.1% identity) and Tk-subtilisin (PDB ID: 2Z2Y, green, 28.9% identity). SUB1 secondary structure elements are shown above its sequence (spirals, α and 3_10_ (η)-helices; arrows, β-strands), the three insertion loops are indicated with loop 1 (Leu388–Tyr411) containing a double calcium site unique to SUB1, loop 2 (Ser522–Lys533) originating from the surface-exposed disulphide bridge Cys521–Cys534, and the smaller and more polar loop 3 (Lys561–Tyr568). Cys residues are highlighted in yellow and disulfides indicated in black. The catalytic triad is indicated with black squares and the positions of bound calcium ions indicated with blue, magenta or green dots in accord with the corresponding sequence. (**c**,**d**) Superimposition of rPfSUB1_cat_ (grey cartoon) with thermophilic subtilisins thermitase (PDB ID: 1THM, magenta) and Tk-subtilisin (PDB ID: 2Z2Y, green). The common high-affinity calcium-binding site (Ca1) is shown. Calcium ions bound to rPfSUB1_cat_ and the thermophilic subtilisins are shown in blue, magenta and green. The PfSUB1 catalytic triad is in black sticks. Insertion loop 1, loop 2 and loop 3 are shown. The Tk-subtilisin insertion loop that contains four bound Ca^2+^ ions and resembles the PfSUB1 loop 1 is indicated.

**Figure 2:**
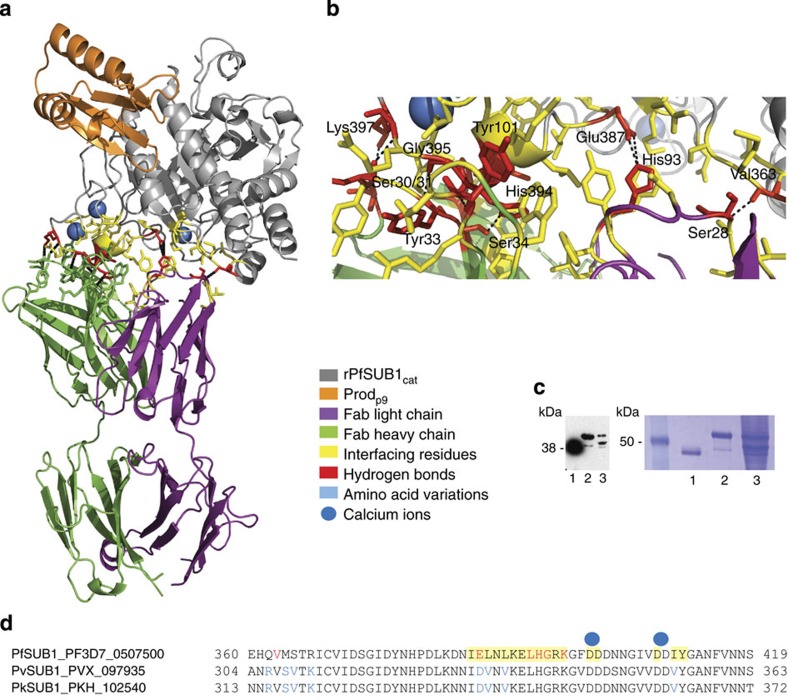
Structure of the rPfSUB1_cat_–Prod_p9_–Fab complex and the NIMP.M7 epitope. (**a**) Cartoon representation of the crystal asymmetric unit content with rPfSUB1_cat_ shown in grey, Prod_p9_ in orange, and the mAb NIMP.M7 Fab light and heavy chains in purple and green, respectively. The three bound Ca^2+^ ions are represented as blue spheres. The NIMP.M7 epitope, located on the calcium-binding PfSUB1 loop 1 (see Fig. 1) is shown with interfacing residues in yellow, or red where hydrogen bonds are formed. (**b**) Zoomed view of the interface region, showing the side chains of the residues involved, with hydrogen bonds represented as black dashed lines and residues (red) involved in hydrogen bonds labelled. (**c**) The mAb NIMP.M7 epitope is conserved in SUB1 orthologs as determined by western blot against rPfSUB1_cat_ (lane 1), recombinant *P. vivax* SUB1 (lane 2) and recombinant *P. knowlesi* SUB1 (lane 3), under reducing conditions, although the interaction appears weaker for *P. vivax* and *knowlesi* proteins (a Coomassie-stained SDS–PAGE gel is shown alongside to show approximately equal loading of the proteins). (**d**) Sequence alignment of PfSUB1 residues 360–419 with the corresponding sequence from the *P. vivax* and *P. knowlesi* orthologues (PlasmoDB gene ID PF3D7_0507500, PVX_097935 and PKH_102540, respectively; see http://www.plasmodb.org/plasmo/) shows that the loop 1 insertion (Leu388–Tyr411) is conserved, as are the calcium-coordinating residues and those involved in the NIMP.M7 epitope. The residues involved in the NIMP.M7 interaction are highlighted in yellow, or in red when hydrogen bonds are present. Amino-acid differences between the SUB1 orthologs are indicated in blue.

The structure of the rPfSUB1_cat_–Prod_p9_–Fab complex was solved by molecular replacement at 2.25 Å with refined *R* factors *R*/*R*_free_ of 0.19/0.23 (Figs 1a,b and 2 and Table 1). Two data sets (with and without 20 mM CaCl_2_ at the crystallization stage) were collected to attempt to identify potential additional medium to low-affinity calcium sites. The rPfSUB1_cat_ catalytic domain comprises 336 residues (Tyr333–Lys668) with 324 residues well defined in density, overall dimensions of 56 × 35 × 45 Å, and an α/β-fold typical of the S8 subtilisin family. The twisted central parallel β-sheet contains six strands (β1, Arg367–Asp372; β2, Lys454–Lys459; β4, Met486–Gly489; β5, Phe514–Ser517; β6, Val553–Lys561 and β8, Leu583–Pro586) with the addition of a small antiparallel strand (β7, Tyr568–Leu570) that interacts with β6 to form a beta hairpin absent from other subtilisins. The catalytic domain contains a total of 14 helical elements with nine main α-helices (α1, Trp344–Ser349; α2, Gln355–His361; α3: Leu390–His394; α4, His428–Ser437; α5, Leu469–Arg482; α6, Gly498–Lys510; α7, Thr605–Ile622; α8, Tyr628–Ser638 and α9, Ile657–Ser667). The catalytic triad is structurally conserved with Asp372 at the C-terminus of β1, and His428 and Ser606 at the N-termini of α-helices α4 and α7, respectively. A striking feature is the large number of aromatic residues (a total of 44) that are found at or near the surface of the molecule and involved in the formation of aromatic clusters (Supplementary Fig. 1). Despite its primary sequence similarity to the subfamily S8A subtilisins^14^, the closest fold relative of rPfSUB1_cat_ as calculated using DALI^15^ is the subfamily S8B thermostable subtilisin thermitase (PDB ID: 1THM; *Z*-score of 41, Cα root mean squared deviation (r.m.s.d.) value of 0.71 Å) from *Thermoactinomyces vulgaris*^16^ (Fig. 1b,c). PfSUB1 possesses the high-affinity calcium-binding site (Ca1) found in thermitase and other subtilisins (Fig. 1, Supplementary Fig. 2a). Dominant structural differences comprise three surface insertion loops, denoted loop 1 (Leu388–Tyr411), loop 2 (Ser522–Lys533) and loop 3 (Lys561–Tyr568) with loop 1 containing two additional Ca^2+^ ions in a binding site that has no equivalent in any other subtilisin and which also constitutes the NIMP.M7 Fab epitope (Fig. 2). A somewhat similar feature is observed in Tk-subtilisin from *Thermococcus kodakaraensis*^17^ (Cα r.m.s.d. value of 1.48 Å) with a different insertion loop that allows the high-affinity binding of four Ca^2+^ ions (Fig. 1d, Supplementary Fig. 2b). In both Tk-subtilisin and PfSUB1, this calcium-binding loop is essential for protein folding^12^,^18^. As rPfSUB1_cat_–Prod_p9_–Fab was initially crystallized without added calcium, all three calcium-binding sites are likely of high affinity (Fig. 1, Supplementary Fig. 2c, Supplementary Table 1). No additional calcium binding was identified in crystals grown in 20 mM CaCl_2_ (rPfSUB1_cat_–ca), despite the presence of other potential calcium-binding loops (Gly412–Met423 and Ile375–His379/Pro595–Ser598) that are conserved in the thermophilic enzymes (Supplementary Fig. 2a,b) and despite the fact that, as previously observed for rPfSUB1_p54_^13^, rPfSUB1_cat_ enzyme activity exhibits calcium dependence, with optimal activity in the range ~40–500 μM calcium (Supplementary Fig. 2d). Calcium addition has an activating effect and also stabilizes the enzyme fold as determined by a thermofluor assay, which demonstrated that the melting temperature of the enzyme increases by 2–3 °C with increasing calcium concentration (Supplementary Fig. 2e). In contrast, the chelating agent EDTA dramatically destabilized the rPfSUB1_cat_ fold with a 12 °C melting temperature drop, indicating that calcium binding is essential for its fold integrity.

**Figure 3:**
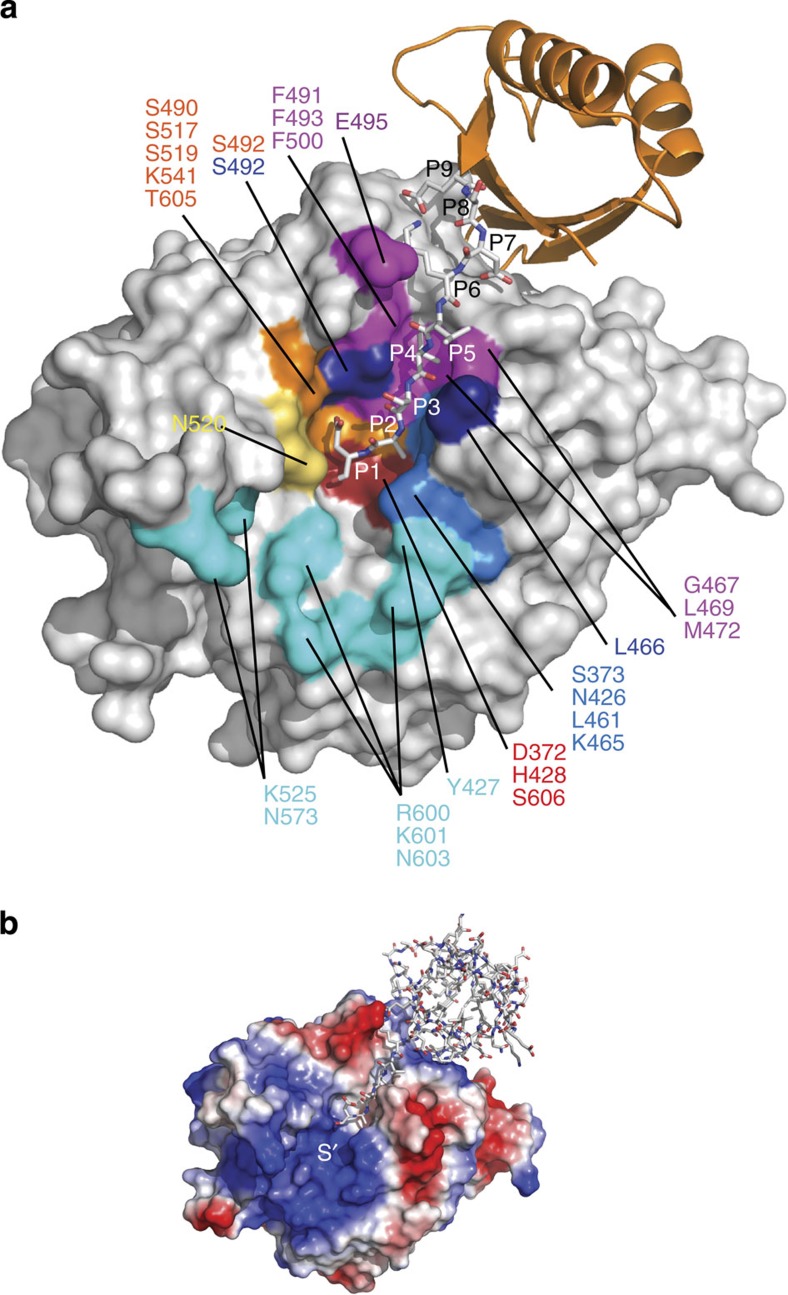
Overall architecture of the PfSUB1 active site. (**a**) Molecular surface representation of rPfSUB1_cat_–ca (structure with CaCl_2_) with its polar active site pocket S1 (orange), the constricted S2 pocket (blue), the S3 pocket (dark blue) and the highly hydrophobic S4 pocket (magenta). The large basic S' surface pocket is indicated (cyan). Residues belonging to the active site pockets are indicated and coloured accordingly, with the catalytic triad residues (Asp372, His428 and Ser606) in red and the oxyanion hole partner Asn520 in yellow. Prod_p9_ P9–P1 residues (E209-SDKLVSA-D217) are shown as white sticks and the remainder of the molecule as an orange cartoon. (**b**) Electrostatic molecular surface potential of rPfSUB1_cat_, with Prod_p9_ represented as white sticks and illustrating the highly positively charged nature of the S' pocket. Acidic prime-side substrate residues are likely stabilized via complementary electrostatic interactions with the basic S' pocket residues.

The bound prodomain fragment, Prod_p9_ is truncated at its amino (N) terminus as a result of the chymotrypsin digestion step used during purification. Prod_p9_ comprises 81 residues, with Lys137–Glu209 forming a compact globular lobe appended by a long carboxy (C)-terminal ‘stalk’ (Ser210–Asp217) (Figs 1a and 3a). Prod_p9_ resembles other subtilisin prodomains^17^,^19^,^20^ (Supplementary Fig. 3a,b) and is made up of four antiparallel β-strands (Leu140–Glu144, Gly170–Leu175, Asn179–Leu185 and Ala206–Ser210) that pack extensively against the two parallel α-helices α5 and α6 of rPfSUB1_cat_, as well as two surface antiparallel α-helices (Pro160–Lys169, Asp191–Lys204) that face the crystal solvent channel. The closest fold relative of Prod_p9_ as shown by DALI is TgMIC5 (*Z*-score of 5.9) from the related protozoan *Toxoplasma gondii*^21^ (Supplementary Fig. 3c). TgMIC5 is not expressed as a cognate subtilisin prodomain, but as a stand-alone prodomain mimic that regulates the activity of a distinct subtilisin-like enzyme, TgSUB1. The structural similarity between Prod_p9_ and TgMIC5 is remarkable given their low (12%) sequence identity, and only residues corresponding to P4 (Val) and P7 (Asp) are conserved within the C-terminal strand. The P9 residue of Prod_p9_, a conserved Glu in other subtilisin prodomains that caps a contacting helix of the catalytic domain (Glu209 in PfSUB1), is replaced by a Gly in TgMIC5, a substitution predicted to impact on the stability of the MIC5–TgSUB1complex (Supplementary Fig. 3d), perhaps enabling reversible interactions.

### PfSUB1 binds both prime and non-prime substrate residues

**Figure 4:**
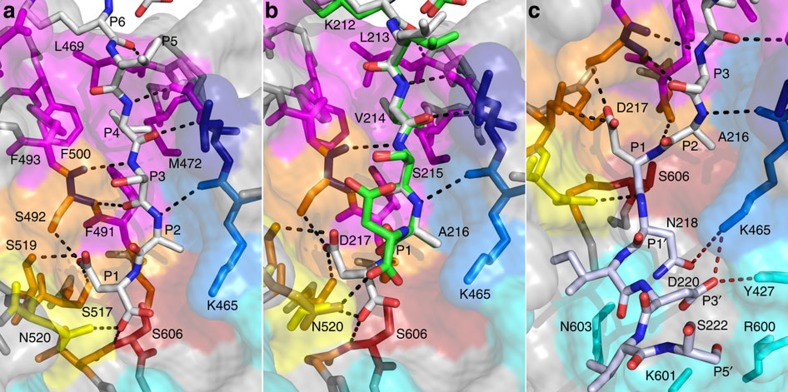
Structure of the rPfSUB1_cat_ active site enables interactions with both prime and non-prime-side substrate residues. (**a**) Zoomed view of rPfSUB1_cat_–ca–Prod_p9_ interactions in the active site. Canonical binding is indicated by black dashed lines involving main-chain residues. The P1 residue Asp217 interacts with oxyanion hole partner Asn520 and residues Ser517, Ser519 and Ser492 at the bottom of the polar S1 pocket. The catalytic Ser606 is indicated in red. The P2 (Ala216) space is restricted by the side chain of Lys465, which forms the side of the compact S2 pocket. The P3 residue (Ser215) faces the solvent and P4 (Val214) is stabilized by Met472, Phe491, Phe493 and Phe500, which shape the hydrophobic S4 pocket. (**b**) Superimposition of rPfSUB1_cat_–ca–Prod_p9_ (white sticks) with rPfSUB1_cat_ Prod_p9_ (P1–P7) (green sticks), illustrating the increased stabilization of Prod_p9_ P1 in the S1 pocket (interacting residues Ser517, Ser519, Ser594) in the calcium-supplemented structure. The Asn520 side chain is shown darker in rPfSUB1_cat_. Hydrogen bonds are indicated (black dashed lines). (**c**) Docking of the decapeptide LVSAD↓NIDIS (scissile bond indicated by a downward-pointing arrow) corresponding to the P5–P5′ residues of the endogenous PfSUB1 prodomain cleavage site, into the rPfSUB1_cat_ active site, based on the crystal structure of the rPfSUB1_cat_–ca–Prod_p9_ complex. Important prime-side interactions likely involve P1′ and P3′ with Lys465 and Tyr427 (hydrogen bonds are shown as red dashed lines).

The C-terminal stalk of Prod_p9_ binds in the active site groove in a substrate-like manner (Figs 1a and 3a). Canonical main-chain interactions involving Prod_p9_ residues P2 (Ala216) to P6 (Lys212) and rPfSUB1_cat_ residues, Lys465–Leu469, result in the formation of a small antiparallel two-stranded β-sheet, as observed in many subtilisins. However, the active site groove of PfSUB1 is narrower and deeper than that of other subtilisins. It lies between the two loops connecting the structural segments β2–α5 and β4–α6. The S1, S2 and S4 pockets are clearly delineated by side chain residues that provide walls to the cavity (Figs 3a and 4a). S1 is predominantly polar with Ser490, Ser517, Asn520, Thr605 and Ser606 at the bottom of the pocket and the side chains of Ser492 and Ser519 at the side, with Lys541 on the cavity top opening. In the calcium-supplemented rPfSUB1_cat_–ca structure, the side chain of the Prod_p9_ P1 residue Asp217 is plugged into the S1 cavity with hydrogen bonds involving Ser517, Ser519 and Ser492 (Fig. 4a). Importantly, the Asp217 P1 carbonyl is stabilized by a short (2.59 Å) hydrogen bond with oxyanion hole partner Asn520. In this structure, further stabilization of the P1 Asp217 is assured by main-chain interactions involving Ser490 and Ser606, extending canonical interactions with the P1 residue (Supplementary Table 2). By contrast, in the thermally less stable rPfSUB1_cat_ structure (Cα r.m.s.d. value of 0.64 Å Table 1), both the P1 side chain stabilization in the S1 pocket and the backbone interactions are lost, leaving a single weaker hydrogen bond with Asn520 (Fig. 4b). The S2 pocket is clearly constricted by the aliphatic hydrocarbon side chain of Lys465, which runs along the pocket, and Leu461 at the bottom, consistent with previous observations of a restriction to Gly or Ala at the P2 position in known physiological PfSUB1 substrates^3^,^6^,^7^,^10^,^13^. The S4 pocket, which is highly hydrophobic, is constituted by Gly467, Leu469, Met472, Phe491, Phe493, Phe500 in the groove and Glu495 at the side entrance of the pocket. The Prod_p9_ P4 residue Val214 is neatly sandwiched between Phe491, Phe493, Phe500 and Met472 (Fig. 4a,b). The less prominent S3 pocket consists of main-chain interactions involving Ser492 with the Prod_p9_ P3 residue Ser215, which faces the solvent.

The structure of the S′ pocket reveals the nature of the interactions that determine the preference for prime-side acidic or hydroxyl-containing residues. The pocket is delimited on one side by the insertion loop 2 (Ser522–Lys533) with the Cys521–Cys534 disulphide bond exposing Cys521 to the active site entrance and Lys525 at the top of the loop, and on the other side by the surface-exposed residues Arg600, Lys601 and Asn603 preceding the active site Ser606. Based on previous docking of dodecapeptide substrates into the active site of a PfSUB1 homology model^10^,^22^, we extended *in silico* the Prod_p9_ C-terminus from P1 to P5′ in the active site of rPfSUB1_cat_ (Fig. 4c). This suggested that rPfSUB1_cat_ residue Lys465, which runs along the S2 pocket, makes additional contributions to the unusual basicity of the S′ pocket and to potential interactions with P' side substrate residues^8^ (Fig. 3a,b, Fig. 4c). In addition, the side chain hydroxyl of Tyr427, which immediately precedes the catalytic His and lies adjacent to the Lys465 amino group, is likely of importance in the selectivity of PfSUB1, assisting Lys465 in stabilizing acidic or polar residues at the P1′ and P3′ positions (Fig. 4c). Although the Tyr427 side chain is well defined in density, it constitutes the only Ramachandran outlier of the structure, possibly adopting a specific conformation for optimal substrate interaction at the P′ side.

### A labile disulphide switch regulates PfSUB1 catalysis

**Figure 5:**
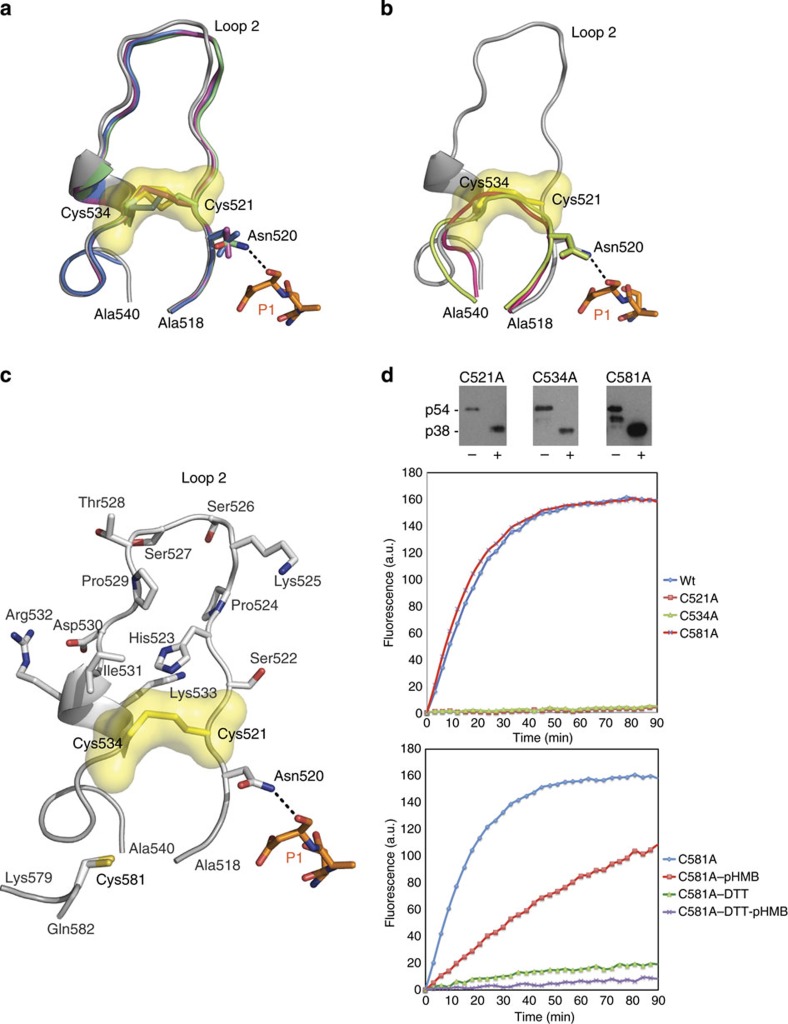
A regulatory redox switch in rPfSUB1_cat_. (**a**) Cartoon view of rPfSUB1_cat_ loop 2 in grey (Ala518–Ala540; the remainder of the molecule is removed for clarity) superimposed on molecular models of the corresponding sequence in *P. vivax* SUB1 (blue), *P. knowlesi* SUB1 (green) and *P. berghei* SUB1 (magenta). Most side chains are removed for clarity. Loop 2 and the Cys521–Cys534 disulphide are conserved in the SUB1 orthologs. The hydrogen bond between the oxyanion hole Asn520 that stabilizes the carbonyl of the Prod_p9_ P1 Asp217 is shown (black dashed line). Prod_p9_ P1–P3 residues are shown as orange sticks. (**b**) Cartoon view of rPfSUB1_cat_ loop 2 as in **a** superimposed on thermitase (PDB ID: 1THM, pink) and Tk-subtilisin (PDB ID: 2Z2Y, green). The oxyanion hole partner side chains (Asn520 in PfSUB1) superimpose perfectly when the rPfSUB1 Cys521–Cys534 disulphide is formed. (**c**) Cartoon view of rPfSUB1_cat_ loop 2, showing side chains as labelled sticks, the labile Cys521–Cys534 disulphide (yellow) and the buried free Cys581, <12 Å away from the disulphide. Alternative conformations of Cys581 are shown. (**d**) Top: western blot analysis (probed with a rabbit antiserum specific for PfSUB1) of purified rPfSUB1 mutants C521A, C534A, C581A before (−) or after (+) digestion with chymotrypsin. The chymotrypsin-digested forms of the mutant proteins were used for kinetic assays shown below. Middle: progress curves depicting hydrolysis of fluorogenic peptide substrate SERA4st1F-6R12 (0.1 μM final) by wt rPfSUB1_cat_ and the various mutants. Initial hydrolysis rates for C521A and C534A are ~1% of those for wt PfSUB1_cat_ or the C581A mutant. Each purified protein was diluted in reaction buffer (20 mM Tris–HCl pH 8.2, 150 mM NaCl, 12 mM CaCl_2_, 25 mM CHAPS) to a final concentration of 0.2 μM for the assay. Points on the plots are mean averages of duplicate measurements from three independent experiments. Bottom: progress curves showing the effects of pHMB alone (2 mM) or DTT alone (5 mM) or both reagents together on hydrolytic activity of the C581A mutant. The observed inhibition is consistent with the redox-sensitive nature of the Cys521–Cys524 disulphide. Assay conditions are as in the middle plot.

The rPfSUB1_cat_ structure contains a total of seven Cys residues and three disulphide bridges. The free sulfhydryl of Cys581 is buried and located less than 12 Å from the Cys521–Cys534 disulphide that restrains loop 2, a prominent structure that is conserved in *Plasmodium* SUB1 orthologs but absent from thermophilic subtilisins (Fig. 5a,b). The PfSUB1 oxyanion hole partner Asn520, which requires an optimum side chain orientation to stabilize the carbonyl of the Prod_p9_ P1 residue Asp217 during catalysis, immediately precedes Cys521. The PfSUB1 Asn520 side chain superimposes perfectly with the oxyanion hole partner of the thermophilic enzymes when the Cys521–Cys534 disulphide is formed (Fig. 5b), indicating that in PfSUB1 this structural setting corresponds to an active enzyme. However, the Cys521–Cys534 disulphide bond, which is partially exposed to the solvent, is clearly unstable in both rPfSUB1_cat_ (C521 Sγ/C534Sγ: 74.27 Å^2^/56.14 Å^2^) and rPfSUB1_cat_–ca (C521Sγ/C534Sγ: 58.60 Å^2^/24.22 Å^2^), though less so in the calcium-supplemented structure where there is also improved stabilization of the P1 carbonyl group by Asn520 and of the P1 side chain in the S1 pocket (Fig. 4b). The two other disulphides in rPfSUB1_cat_ (Cys369–Cys479 and Cys458–Cys475) are buried and likely to act purely as structural elements (Fig. 1a). We have previously shown^13^ that PfSUB1 is sensitive to inhibition by both *para*-hydroxymercuribenzoate (pHMB), an organomercurial that covalently modifies cysteine sulphydryls, and the reducing agent DTT at a concentration (10 mM) that would not be expected to affect PfSUB1 fold integrity. To examine this inhibition in more detail, we analysed pHMB-treated and TCEP-reduced rPfSUB1_cat_ by mass spectrometry. This confirmed that inhibition is associated with a change in the state of one or more rPfSUB1_cat_ Cys residues (Supplementary Fig. 4). Binding of pHMB to rPfSUB1_cat_ was incomplete unless the enzyme was pretreated with 1 mM TCEP, when binding reached 100%, with a single Hg substitution (Supplementary Fig. 4b). This is consistent with rPfSUB1_cat_ existing in solution as a redox-sensitive mixture of oxidized and reduced forms. Liquid chromatography–mass spectrometry/mass spectrometry analysis (Supplementary Table 3) showed that pHMB does not react with Cys581, but instead modifies Cys521 or Cys534. This likely leads to enzyme inhibition due to a structural change in the vicinity of the active site or by abolishing formation of the Cys521–Cys534 disulphide bridge, either event likely to have a detrimental effect on the geometry of the oxyanion hole Asn520.

To confirm the importance for enzyme activity of the Cys521–Cys534 disulphide bridge, three rPfSUB1 mutants were produced, each containing a single amino-acid substitution of either Cys521, Cys534 or Cys581. All three mutant proteins (called C521A, C534A and C581A) underwent correct processing to the p54 form during recombinant expression in baculovirus-infected insect cells, demonstrating a retained capacity to undergo autocatalytic maturation (Fig. 5d). However, kinetic analysis of the purified, chymotrypsin–treated recombinant proteins showed substantially decreased hydrolytic activity for the C521A and C534A mutants (Fig. 5d), consistent with a critical role for the Cys521–Cys534 disulphide bridge in catalytic activity. In contrast, the C581A mutant behaved kinetically like the wild-type enzyme and moreover retained sensitivity to both pHMB and reducing conditions, demonstrating the redox-sensitive nature of the Cys521–Cys534 disulphide bridge (Fig. 5d). The instability of the Cys521–Cys534 bond, together with the evident degree of stress in the entire loop 2 (which is flexible with poor electronic density), and the dependence of PfSUB1 activity on the state of the cysteines involved in the bond, are collectively characteristics of a redox-sensitive disulphide switch^23^,^24^.

## Discussion

Successful crystallization of recombinant PfSUB1 proved a substantial technical challenge, requiring chymotryptic conversion of the enzyme to a soluble core and production of a complex with a monoclonal antibody Fab fragment. Inevitably, therefore, our rPfSUB1_cat_–Prod_p9_–Fab structure does not provide a complete picture of the molecular structure of the native mature enzyme. Nonetheless, it provides important and useful mechanistic insights into the substrate specificity and mode of regulation of an essential malarial protease. The overall fold of the PfSUB1 structure resembles thermophilic subtilisins, both in its aromatic content and in the presence of several tightly bound Ca^2+^ ions. rPfSUB1_cat_ contains an astonishing 44 aromatic residues, many more than in bacterial subtilisins (16 in subtilisin BPN' and 22 in subtilisin Carlsberg) and an even higher number than in thermophilic subtilisins (24 in thermitase and 34 in Tk-subtilisin). As in the thermophilic enzymes, these residues predominantly lie near the surface of the molecule and organize into aromatic clusters that contribute to the overall stability of the enzyme core. rPfSUB1_cat_ also contains three bound Ca^2+^ ions, likely bound with high affinity as no calcium was added during purification or the initial crystallization procedures. Bound Ca^2+^ ions are found in all subtilisins except plant subtilisins^25^, and often play an important role in maturation, activity and stability^26^,^27^. Subfamily S8A subtilisins generally have two Ca^2+^ sites respectively of high and low affinity^8^,^27^. These sites are shared with subfamily S8B members such as thermitase, which also has a third site of medium affinity^28^. In PfSUB1, even though no additional calcium sites were identified in the complex crystallized in the presence of 20 mM CaCl_2_, calcium enhanced both enzyme activity and stability as measured by thermofluor assay. Incorporation of additional Ca^2+^ ions may have been impaired in our crystal structure due to the presence of the Fab in the crystallized complex; its epitope is located on loop 1, which contains a double calcium-binding site when the Fab is bound and that could possibly bind additional Ca^2+^ ions in the absence of the Fab. Under physiological conditions, calcium is likely important for optimal PfSUB1 activity against its authentic substrates *in vivo*, as the PV is a relatively calcium-rich environment with calculated concentrations in the micromolar range^29^.

The cognate interaction between the N-terminally truncated prodomain Prod_p9_ and rPfSUB1_cat_ in the crystallized complex was instrumental to the identification of the active site residues directly involved in substrate binding. The resulting structure reveals PfSUB1 as an unusual subtilisin with a hydrophobic S4 pocket, a small S2 pocket, a highly polar S1 pocket and an extended, basic S' pocket that plays an important role in substrate stabilization. Previous experimental work has shown that replacement of the P3′ Asp with Ala in a small synthetic substrate has a significant effect on catalysis, with an ~50% reduction in cleavage efficiency^6^. Similarly, synthetic peptide substrates entirely lacking a prime-side extension, or with both P1′ and P3′ residues substituted with Ala, were cleaved inefficiently or not at all, indicating a requirement for at least 3 prime side residues for efficient catalysis, with P1′ and/or P3′ being acidic or polar^10^. Our structural data now explain those findings; in both our rPfSUB1_cat_ crystal structures, the substrate P1′ and P3′ side chains can interact directly with the amino group of Lys465 and the hydroxyl of Tyr427. The observed substrate stabilization at the P1′ and P3′ positions is also in complete agreement with the high frequency of acidic and/or hydroxyl-containing side chains in the prime-side positions of physiological PfSUB1 substrates. While limited interactions with prime-side residues have been described previously in S8A subtilisins^30^, to our knowledge PfSUB1 is the first member of the subfamily to display such extensive prime-side substrate requirements. This feature could be exploited in the design of selective drug-like inhibitors, an approach currently being explored^10^.

The presence of calcium in the crystallization buffer led to a rPfSUB1_cat_ crystal structure with increased stabilization of the Prod_p9_ P1 residue (Asp217) in the enzyme active site S1 pocket. This appears to be directly related to the stability of the solvent-exposed Cys521–Cys534 disulphide bridge in the vicinity of the S1 pocket, one residue away from the oxyanion hole partner Asn520. In the crystal structure without added calcium, a larger proportion of the Cys521–534 disulphide bond is reduced resulting in a modification of the oxyanion hole Asn520 geometry and a decrease in P1 stabilization. In solution, we also observed the presence of a mixture of both the reduced and oxidized forms of Cys521–Cys534, with loss of protease activity on complete reduction of the disulphide bridge. Disruption of this bridge in the C521A and C534A rPfSUB1 mutants severely impacted on protease activity, confirming the importance of the disulphide in PfSUB1 catalysis. Collectively, these results clearly illustrate the labile nature of the Cys521–Cys534 disulphide bond and suggest that its redox status may be influenced by environmental conditions within the parasite. Compartmentalization of redox conditions in the intraerythrocytic parasite has been demonstrated, and while the parasite cytosol is highly reducing^31^ it is generally considered that the PV lumen maintains an oxidising, ‘extracellular-like’ environment. In contrast, little is known of the redox status of the various secretory organelles of the parasite, including the exonemes where SUB1 is stored before its discharge into the PV. If, like the parasite cytosol, the exonemes are also a reducing environment, this might be predicted to maintain the stored SUB1 in an enzymatically inactive form due to reduction of the Cys521–Cys534 disulphide bridge, preventing autolysis and/or inappropriate proteolytic cleavage of other exoneme components. On discharge of the enzyme into the PV, oxidative reconstitution of the Cys521–Cys534 disulphide would effectively activate the enzyme, providing a simple mechanism for regulating SUB1 activity in the parasite. All seven Cys residues in the PfSUB1 catalytic domain are conserved in all SUB1 orthologs^12^, implying that the labile nature of the Cys521–Cys534 disulphide bond and its capacity to modulate SUB1 activity is conserved in all *Plasmodium* species. The relatively calcium-rich environment of the PV may further influence the stability of the Cys521–Cys534 disulphide as seen in our rPfSUB1_cat_–ca crystal structure, aiding protease activity. Non-catalytic regulatory cysteines are becoming increasingly recognized for their importance in controlling enzyme function^23^. Our work shows that SUB1 likely belong to this class of enzymes.

In conclusion, our study provides a description of the architecture of the PfSUB1 active site cleft at the atomic level, essential for directed design of selective inhibitors and rational drug development. In addition, we present evidence for the first functional redox switch within the subtilisin family. The accessibility and reactivity of Cys521 offers potential for the design of new chemotherapeutic agents to modify the redox equilibrium of PfSUB1 and thereby block progression of the parasite life cycle.

## Methods

### Generation of antibodies and Fab preparation

A polyclonal rabbit antiserum raised against recombinant PfSUB1 (ref. 13) was used for western blot analysis at a dilution of 1:1,000. Purification of mAb NIMP.M7 (ref. 4) was by affinity chromatography using Protein G Sepharose (GE Healthcare, UK). Immobilized Ficin was used to prepare Fab fragments from purified NIMP.M7 according to the manufacturer’s instructions (Pierce Mouse IgG1 Fab and F(ab′)2 Preparation Kit, Thermo Scientific). Fab fragments were generated in the presence of 25 mM cysteine and 0.25 ml of the settled Ficin resin, and the digestion reaction incubated for 4 h at 37 °C, followed by purification on NAb Protein A Spin Columns. The Fab fraction was further purified on a HiLoad 26/60 Superdex 200 prep-grade column (GE Healthcare, UK) before being used for complex formation with rPfSUB1_cat_–Prod_p9_.

### Protein expression and purification

Recombinant PfSUB1, PvSUB1 and PkSUB1 were expressed as His6-tagged secreted proteins in baculovirus-infected Tn5 insect cells (Invitrogen) and were purified by two steps of affinity chromatography on Blue Sepharose CL-6B (Sigma) then Ni-NTA agarose (Qiagen), followed by gel filtration on a HiLoad 26/60 Superdex 200 prep-grade column (GE Healthcare)^10^. The purified PfSUB1 was then subjected to limited proteolysis with chymotrypsin to produce rPfSUB1_cat_–Prod_p9_ (ref. 10). The rPfSUB1_cat_–Prod_p9_–NIMP.M7 Fab complex was produced by mixing both components at a 1:2 molar ratio in 20 mM Tris–HCl, 150 mM NaCl pH 8.2 and incubation for 2 h on ice before purification on a HiLoad 26/60 Superdex 200 prep-grade column (GE Healthcare). Amino-acid substitution rPfSUB1 mutants C521A, C534A and C581A were produced using a QuikChange Site-Directed Mutagenesis kit (Agilent Technologies) (sequences of all oligonucleotide primers used in this work are shown in Supplementary Table 4) to modify the pFastBac1–PfSUB1 vector^10^. Expression, purification and chymotrypsin digestion of the mutant proteins was as for wt rPfSUB1^10^ (Supplementary Fig. 5).

### Protease activity and thermofluor assay

Fluorogenic peptide substrate SERA4st1F-6R12 was produced by labelling both the terminal Cys residues of the synthetic *N*-actetylated dodecapeptide Ac-CKITAQDDEESC with 6-iodoacetamido tetramethylrhodamine, followed by purification by reversed-phase HPLC^10^. For assays of SUB1 protease activity, the substrate was diluted from stock solutions in dimethylsulfoxide into reaction buffer (20 mM Tris–HCl pH 8.2, 150 mM NaCl, 12 mM CaCl_2_, 25 mM 3-[(3cholamidopropyl)-dimethylammonio] propanesulfonate) to a final concentration of 0.1 μM, and supplemented with purified recombinant protease. The resulting fluorescence increase was continuously monitored with time at 21 °C using a Cary Eclipse fluorescence spectrophotometer (Varian) equipped with a 96-well microplate reader accessory^10^. For the thermofluor assay, test samples were dispensed into 500 μl PCR tubes (25 μl per tube). In total, 5 μg of rPfSUB1_cat_ was used per assay in 20 mM Tris–HCl pH 8.2, 150 mM NaCl supplemented with different concentrations of EDTA (12.5 and 25 mM) or CaCl_2_ (0.125, 0.25, 0.5, 1, 2 and 5 mM). In total, 2.5 μl of a 50 × Sypro Orange solution (Invitrogen) was added to each tube with mixing and the samples were heated from 25 to 95 °C in 0.5 °C steps of 1 min each in a Mx3005P QPCR system (Agilent Technologies). Excitation/emission filters of 492 and 516 nm were used to monitor the fluorescence increase resulting from binding of the Sypro Orange to exposed hydrophobic regions of the unfolding protein. The midpoint of the protein unfolding transition was defined as the melting temperature *T*m.

### Mass spectrometry

Protein molecular mass was determined using a microTOFQ electrospray mass spectrometer (Bruker Daltonics, Coventry, UK). Desalted protein was injected onto the column in 10% (v/v) acetonitrile, 0.1% (v/v) acetic acid, washed in the same solvent and eluted in 60% (v/v) acetonitrile, 0.1% (v/v) acetic acid. Mass spectra were deconvoluted using maximum entropy software (Bruker Daltonics). Liquid chromatography–mass spectrometry/mass spectrometry of trypsin-digested and acidified protein was performed with an Ultimate 3000 nanoRSLC HPLC (Thermo Scientific). The eluent was introduced into an LTQ Orbitrap Velos Pro (Thermo Scientific) using a Proteon NanoES source and a 30-μm ID stainless steel emitter operated at 2 kV. The Orbitrap was operated in a data-dependent acquisition mode using a survey scan at a resolution of 60,000 from *m/z* 300–1,500, followed by MS/MS in the LTQ of the top 6–12 ions. Raw files were processed using Proteome Discoverer 1.3 (Thermo Scientific).

### Crystal structure determination

Purified rPfSUB1cat–Prodp9–Fab complex was concentrated to 3.5 mg ml^−1^ in 20 mM Tris–HCl pH 8.2, 150 mM NaCl. Crystallization assays were set up at 18 °C, using the vapour diffusion technique and 0.2 μl sitting-drops dispensed by a nanodrop Oryx 8 robot (Douglas Instruments) with protein and reservoir solutions mixed in a 1:1 volume ratio. Initial crystals appeared quickly in the PEGs screen (Qiagen, condition F11) with the reservoir containing 20% PEG3350, 0.2 M ammonium formate±40 mM CaCl_2_. The crystals were crushed to provide a seed stock (Seed Bead, Hampton Research) that generated larger and better diffracting crystals in sitting-drops containing 1 μl of 0.1 M Tris–HCl pH 8.5, 20% PEG3350, 0.4 M ammonium formate±40 mM CaCl_2_ mixed with 1 μl of protein solution. Crystals were cryoprotected in the reservoir solution supplemented with 20% PEG3350 (±20 mM CaCl_2_) before being flash-frozen in liquid nitrogen. The crystals belong the monoclinic space group P2_1_ with unit cell dimensions of 73.50 × 74.79 × 78.85 Å for crystal 1 (rPfSUB1cat, no added CaCl_2_) and *β*=103.33° and 73.34 × 76.03 × 77.88 Å and *β*=102.03° for crystal 2 (rPfSUB1cat–ca, in 20 mM CaCl_2_), with one rPfSUB1cat–Prodp9–Fab complex in the asymmetric unit. Both X-ray data sets were collected at 2.25 Å resolution at the Diamond Light Source (Oxford, UK), crystal 1 on beamline I02 (*λ*=0.9795 Å), crystal 2 on beamline I04-1 (*λ*=0.92 Å) and processed with the XDS package^32^. Space group assignment was done by POINTLESS^33^, and scaling and merging were achieved with SCALA and TRUNCATE^34^. The structure of the rPfSUB1cat–Prodp9–Fab was solved using the PHENIX software suite^35^. Molecular replacement calculations using PDB ID: 1MLC (mouse monoclonal IgG1 Fab D44.1) and 1BH6 (*Bacillus licheniformis*, subtilisin Carslberg) as the search models for the Fab fragments and rPfSUB1cat, respectively, were carried out in the programme AutoMR, identifying a clear solution with a log-likelihood gain of 1747 and good packing. The procedure failed when a third ensemble corresponding to the prodomain fragment (template PDB ID: 1SPB, subtilisin BPN' prosegment) was added. AutoBuild successfully traced the Fab heavy and light chains and the rPfSUB1cat chain with the exception of loop 2 (Ser522–Lys533) and loop 3 (Lys561–Tyr568), which were built manually. The model was initially subjected to rigid-body refinement with an *R*_factor_ of 0.25 and *R*_free_ 0.30. Interactive manual model building with Coot^36^ in combination with phenix.refine, allowed 81 residues of the missing prodomain to be fitted into density. At the end of the refinement procedure, the *R*_factor_ was 0.19 and *R*_free_ 0.23 at 2.25 Å resolution, with 99.88% residues in allowed regions (95% in favored). The isomorphic rPfSUB1cat–ca complex was refined to a *R*_factor_/*R*_free_=0.19/0.23 at 2.26 Å, with 99.88% residues in allowed regions and 95% in favored (Supplementary Fig. 6). The DALI server^15^ was used to search the protein structure database for homologous protein folds. Modelling of PvSUB1, PkSUB1 and PbSUB1 was performed with Modeller v9.3 (ref. 37) using rPfSUB1cat as a template. Docking into the rPfSUB1cat active site of the decapeptide LVSADNIDIS, corresponding to residues P5–P5′ of the endogenous PfSUB1 autoprocessing site^11^, was performed in Coot. The electrostatic molecular surface potential of rPfSUB1cat was calculated in PyMol ( http://www.pymol.org/). Figures were also prepared with PyMol. Amino-acid sequence alignments were performed in ENDscript^38^ ( http://www.endscript.ibcp.fr). Interfaces and chain interactions were calculated in PISA^39^ ( http://www.ebi.ac.uk/msd-srv/prot_int/pistart.html). Crystallographic data are summarized in Table 1.

## 

## Author contributions

C.W.-M. conducted the experiments and wrote the initial manuscript. M.S. produced the antibodies. F.H. carried out the molecular biology and insect cell expression. L.F.H. helped refine the crystallization conditions. P.A.W. was involved in data collection at the Diamond synchrotron. S.A.H. performed ESI–MS and LC–MS/MS mass spectrometry. C.E. helped with the thermofluor assay. M.J.B. contributed to writing the manuscript and supervized the project. G.G.D. initiated this project. This work is dedicated to his memory.

## Additional information

**Accession codes:** Coordinates for the rPfSUB1_cat_–Prod_p9_–Fab complex have been deposited in the Protein Data Bank under accession code 4lvn (crystal 1, without CaCl_2_ in the crystallization buffer) and 4lvo (crystal 2, with CaCl_2_ in the crystallization buffer).

**How to cite this article:** Withers-Martinez, C. *et al.* The malaria parasite egress protease SUB1 is a calcium-dependent redox switch subtilisin. *Nat. Commun.* 5:3726 doi: 10.1038/ncomms4726 (2014).

## Supplementary Material

Supplementary Information Supplementary Figures 1-6, Supplementary Tables 1-4 and Supplementary Reference

## Figures and Tables

**Table 1:** Data collection and refinement statistics for rPfSUB1_cat_–Prod_p9_–Fab complex structures.

	**Crystal 1 (−CaCl_2_)**	**Crystal 2 (+20 mM CaCl_2_)**
*Data collection*		
Space group	P2_1_	P2_1_
*Cell dimensions*		
*a*, *b*, *c* (Å)	73.50, 74.79, 78.85	73.34, 76.03, 77.88
*α, β, γ* (°)	90, 103.33, 90	90, 102.03, 90
Resolution (Å)	30–2.25 (2.33–2.25)*	30–2.26 (2.33–2.26)*
*R*_merge_	0.1077 (0.409)	0.0848 (0.5007)
*I*/σ*I*	7.95 (2.67)	11.69 (2.16)
Completeness (%)	99.59 (98.33)	97.56 (91.69)
Redundancy	3.4 (3.3)	3.4 (3.0)
*Refinement*		
Resolution (Å)	30–2.25	30–2.26
No. reflections	38,909	38,421
*R*_work_/*R*_free_	0.1953/0.2375	0.1913/0.2365
*No. atoms*		
Protein	6,403	6,449
Ligand/ion	5	3
Water	214	201
*B-factors*		
Protein	50.80	34.80
Ligand/ion	46.70	22.70
Water	43.50	30.40
*Root mean squared deviations*		
Bond lengths (Å)	0.004	0.004
Bond angles (°)	0.94	0.90

^*^Values in parentheses are for highest-resolution shell. A single crystal was used for each structure.
