# Supplementary material for: The malaria parasite egress protease SUB1 is a calcium-dependent redox switch subtilisin
Source: Nat Commun. 2014 May 2;5:3726. doi: 10.1038/ncomms4726 (PMC4024747; doi:10.1038/ncomms4726)
Supplement: Supplementary Information — Supplementary Figures 1-6, Supplementary Tables 1-4 and Supplementary Reference [file ncomms4726-s1.pdf]

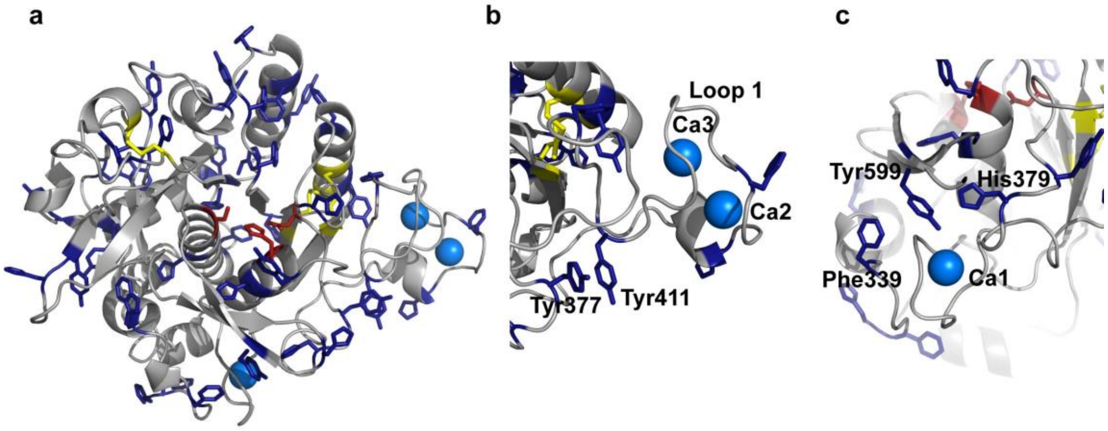

Supplementary Figure 1 | rPfSUB1$_{cat}$ possesses a high content of surface aromatic residues. (**a**) Grey cartoon view of rPfSUB1$_{cat}$ with catalytic triad represented as red sticks, disulfide bonds in yellow and aromatic residues (44 in total) in dark blue, comprising the main T-shaped clusters Phe339/Tyr599, His485/Phe620, Phe339/His379/Tyr599 and Trp344/Tyr564/Tyr568. (**b**) Illustration of a pi sandwich stacking interaction involving Tyr411 and Tyr377 and stabilizing the extended Loop 1 that contains a double calcium binding site. (**c**) Illustration of a surface exposed T-shaped cluster in the vicinity of calcium site 1 and involving Phe339, His379 and Tyr599.

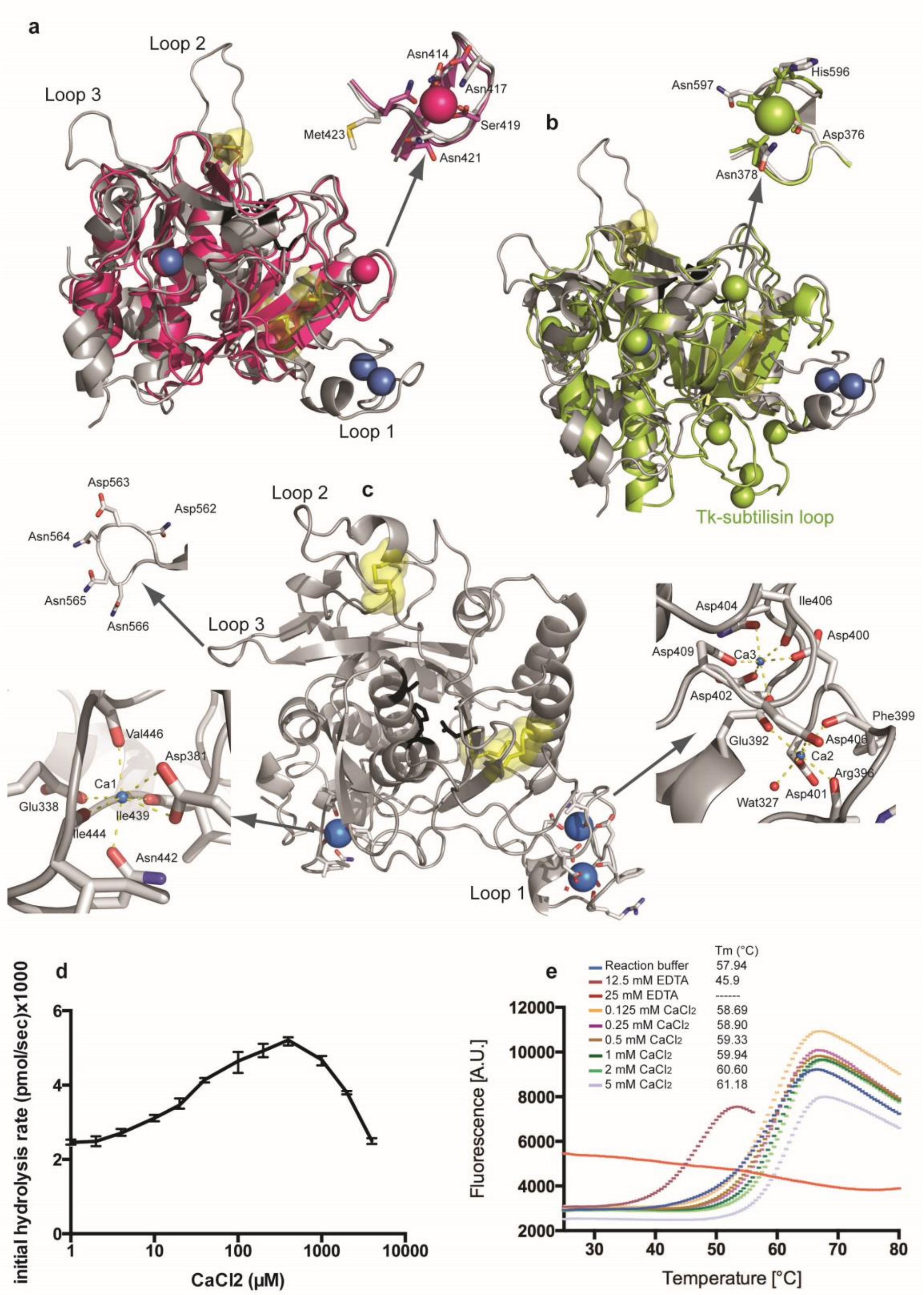

Supplementary Figure 2 | Calcium-binding and calcium-dependence of rPfSUB1$_{cat}$. (ab) Superimposition of rPfSUB1$_{cat}$ (grey cartoon) with thermitase (PDB ID: 1THM, pink) or Tk-subtilisin (PDB ID: 2Z2Y, green). SUB1 insertions Loop 1 (Leu388-Tyr411) containing a double calcium site unique to PfSUB1, Loop 2

(Ser522-Lys533) originating from the surface exposed disulfide bridge Cys521-Cys534 and the smaller and more polar Loop 3 (Lys561-Tyr568) are indicated in (**a**). Catalytic triad residues of $rPfSUB1_{cat}$ are shown as black sticks, and disulfides as transparent yellow spheres. Calcium ions are shown as blue spheres in $rPfSUB1_{cat}$, pink in thermitase and green in Tk-subtilisin. Two structurally conserved potential low affinity calcium-binding sites are shown zoomed with possible coordinator residues labelled. The Tk-subtilisin large insertion loop containing four Ca^{2+} ions and resembling the PfSUB1 Loop1 is also indicated. (**c**) Cartoon view of $rPfSUB1_{cat}$ in grey with the catalytic triad side chains residues as black sticks and the three high affinity calcium ions as blue spheres. The calcium binding site regions are shown zoomed, with bound Ca^{2+} ions depicted as small blue spheres. Coordinating side chain residues are labelled and the hydrogen bonds are shown as dashed yellow lines. A potential low affinity calcium binding site in Loop 3 is also shown zoomed with coordinating residues labelled. (**d**) Calcium-dependence of $rPfSUB1_{cat}$ protease activity, as determined by hydrolysis of the peptide substrate SERA4st1F-6R12 (0.1 μM), in the presence of calcium concentrations ranging from 1 to 5000 μM. The reaction buffer contained 20 mM Tris-HCl pH 8.2, 150 mM NaCl and 25 mM CHAPS; $rPfSUB1_{cat}$ was diluted in the reaction buffer to a final concentration of 0.36 nM for the assay. Data points represent the mean values of triplicate assays, with error bars indicating SD. $CaCl_2$ concentrations are shown on a log scale. (**e**) Thermofluor analysis of $rPfSUB1_{cat}$ performed at various concentrations of EDTA and $CaCl_2$. Corresponding melting temperatures (Tm) are indicated. The chelating agent EDTA (12.5 and 25 mM) has a detrimental effect on enzyme stability indicating that the high affinity calcium binding sites in PfSUB1 are essential for structural integrity.

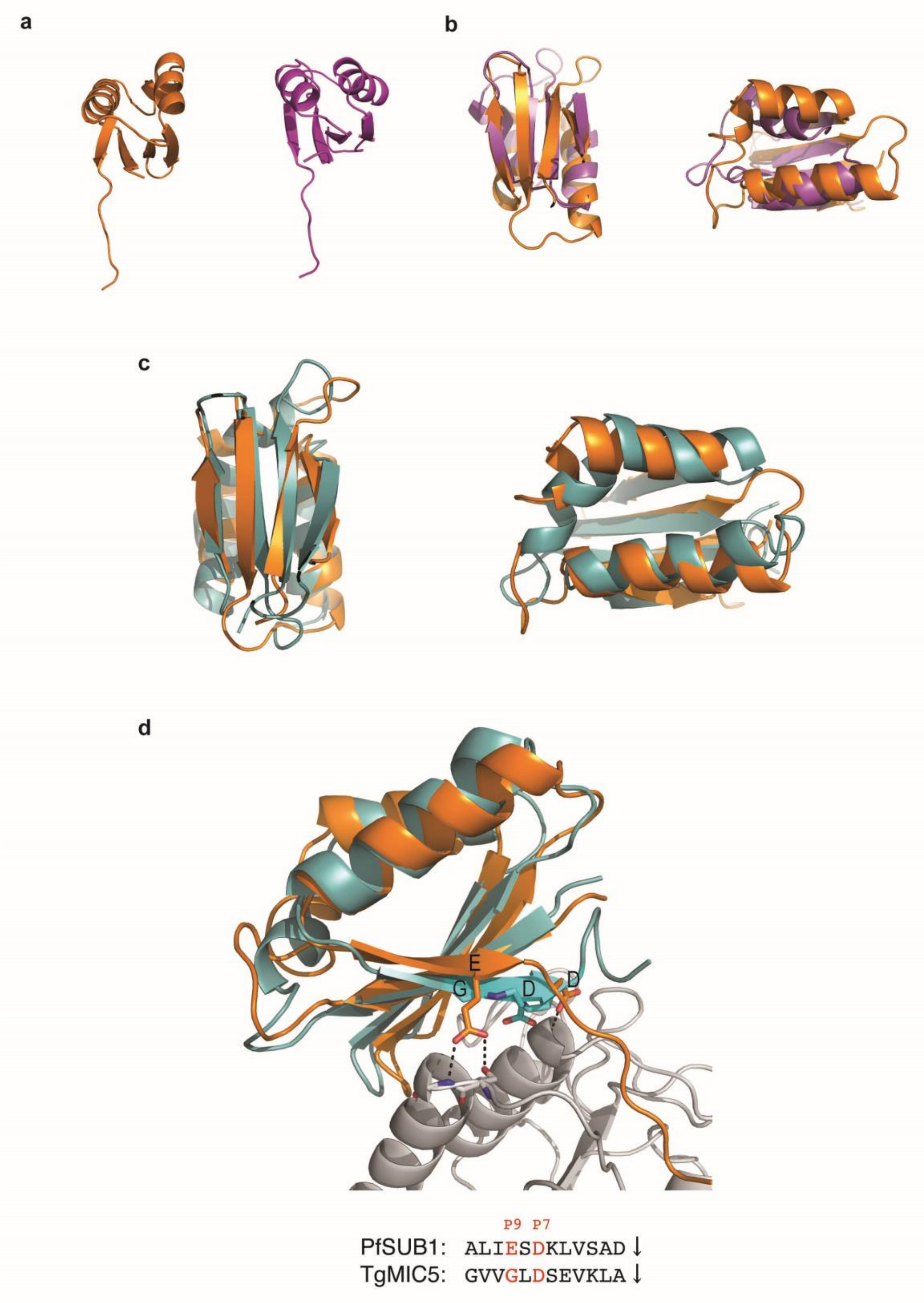

Supplementary Figure 3 | Structural similarity between PfSUB1 Prod$_{p9}$ and TgMIC5. (**ab**) Structural fold comparison between PfSUB1 Prod$_{p9}$ and the subtilisin BPN' prodomain (PDB ID: 1SPB) depicted as orange and purple cartoons respectively, highlighting the regions of the anti-parallel β-sheet and the two surface

α-helices. (**c**) Superimposition of $Prod_{p9}$ (orange) with TgMIC5 (PDB ID: 2LU2) (pale blue) showing the striking similarities within the 4-stranded β-sheet and the two α-helices. (**d**) The same superimposition, shown in the context of the interacting $rPfSUB1_{cat}$ helices (grey) in the $rPfSUB1_{cat}$-$Prod_{p9}$-Fab complex. Differences in the P9 capping residue (Gly in TgMIC5, Glu in $Prod_{p9}$) are indicated. The equivalent P9 residue in all bacterial subtilisins is a Glu. Below is shown an amino acid sequence alignment of the C-terminal 12 residues of $Prod_{p9}$ and TgMIC5 (P12 to P1 relative to the PfSUB1 prodomain cleavage site, indicated by a downward-pointing arrow). The P7 and P9 capping residues are highlighted in red.

a

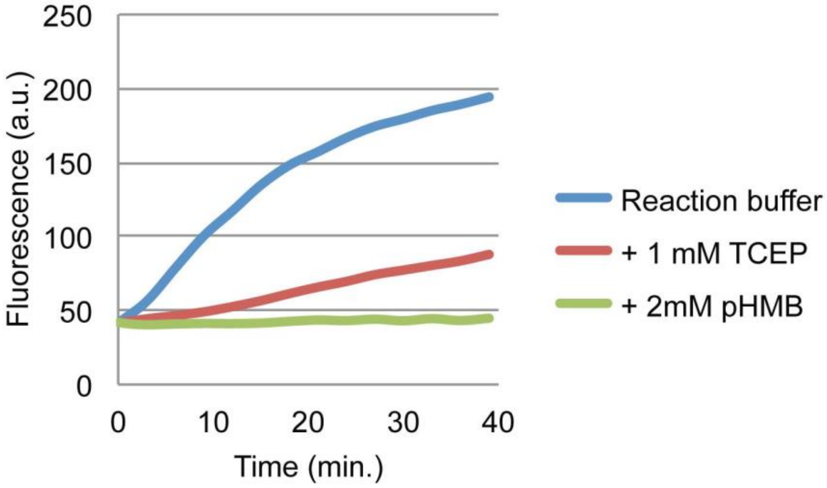

250
200
150
100
50
0
Fluorescence (a.u.)
0
10
20
30
40
Time (min.)
Reaction buffer
+ 1 mM TCEP
+ 2mM pHMB

b

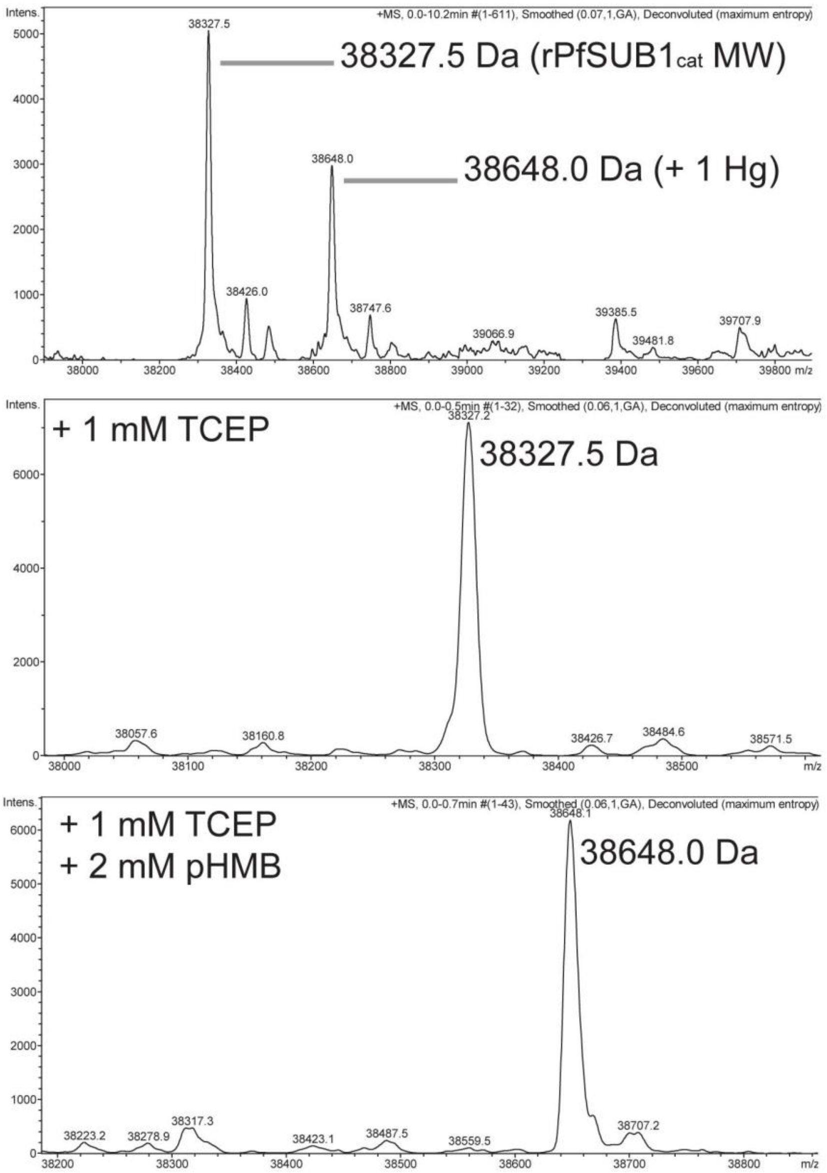

38327.5 Da (rPfSUB1cat MW)
38648.0 Da (+ 1 Hg)
+ 1 mM TCEP
38327.5 Da
+ 1 mM TCEP
+ 2 mM pHMB
38648.0 Da

Supplementary Figure 4 | Enzyme activity assays and mass spectrometry provide evidence for a critical redox-sensitive sulfhydryl residue in rPfSUB1$_{cat}$. (**a**) rPfSUB1$_{cat}$ hydrolytic activity against fluorogenic substrate SERA4st1F-6R12[1], as shown by a time-dependent increase in fluorescence in the presence of protease and substrate. Control levels of activity in reaction buffer only (blue) are inhibited by the reducing agent TCEP (1 mM, red), or pHMB (pHMB, 2 mM, green). Points were recorded in duplicate and averaged. (**b**) ESI-MS spectra. Top: addition of pHMB to untreated rPfSUB1$_{cat}$ results in a mixture of mercury-bound (one Hg atom) and free protein (the predicted molecular mass of rPfSUB1$_{cat}$ with three intact disulfides is 38327.5 Da). Middle: treatment with TCEP alone produces 100% of the 38327.5 Da form of rPfSUB1$_{cat}$, whereas (bottom) exposure of TCEP-treated rPfSUB1$_{cat}$ to pHMB produces 100% of the mercury-bound form.

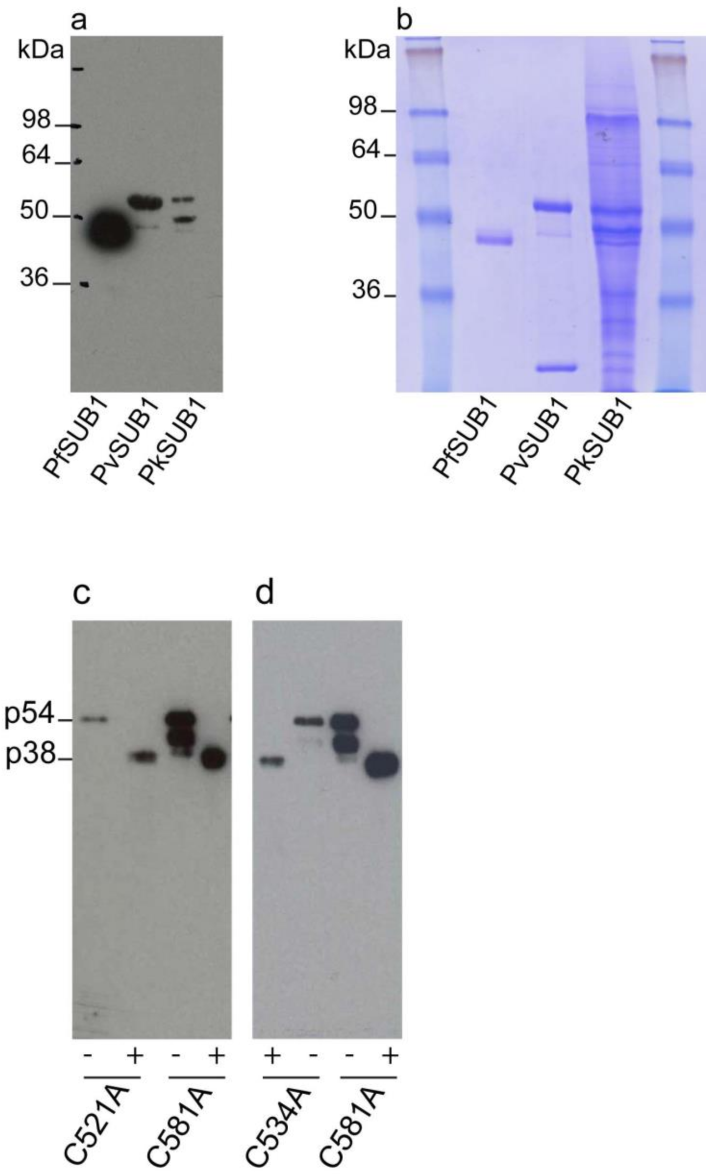

Supplementary Figure 5 | Uncropped forms of the Western blots and Coomassie-stained SDS-PAGE gel images shown in Fig. 2c (ab) and Fig. 5d (cd) of the main manuscript. The positions of migration of molecular mass markers (loaded on both the outermost lanes of the gel) are indicated.

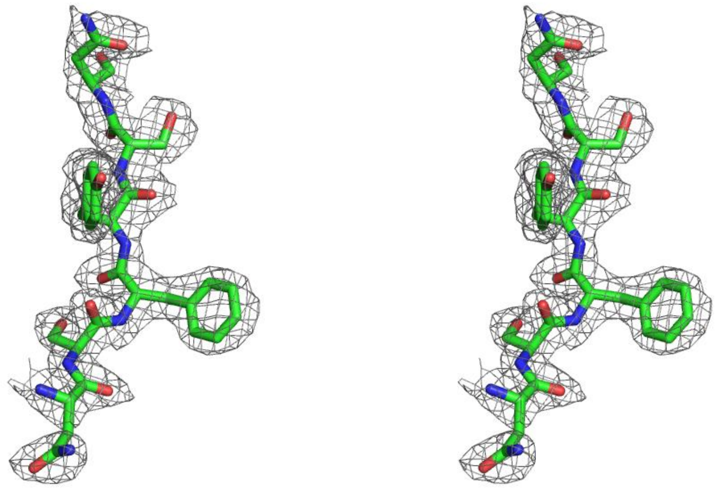

Supplementary Figure 6 | Stereo image of a portion of the PfSUB1$_{cat}$-ca electron density map. The electron density is from a 2FoFc map at 2.26Å resolution, contoured at 1 sigma and including PfSUB1$_{cat}$-ca residues from Asn573 to Asn578, shown as green sticks.

Supplementary Table 1. rPfSUB1$_{cat}$-ca (+ $CaCl_2$) calcium coordination. List of hydrogen bonds.

A:Ca1 701	2.37	A:GLU 338[OE2]
A:Ca1 701	2.43	A:ASP 381[OD1]
A:Ca1 701	2.46	A:ASP 381[OD2]
A:Ca1 701	2.30	A:ILE 439[O]
A:Ca1 701	2.47	A:ASN 442[OD1]
A:Ca1 701	2.35	A:ILE 444[O]
A:Ca1 701	2.46	A:VAL 446[O]
A:Ca2 702	2.37	A:GLU 392[OE1]
A:Ca2 702	2.33	A:ARG 396[O]
A:Ca2 702	2.26	A:PHE 399[O]
A:Ca2 702	2.29	A:ASP 401[OD1]
A:Ca2 702	2.38	A:ASP 408[OD1]
A:Ca2 702	2.34	S:WAT 327
A:Ca3 703	2.38	A:GLU 392[OE2]
A:Ca3 703	2.31	A:ASP 400[OD1]
A:Ca3 703	2.32	A:ASP 402[OD1]
A:Ca3 703	2.47	A:ASN 404[OD1]
A:Ca3 703	2.43	A:ILE 406[O]
A:Ca3 703	2.33	A:ASP 409[OD1]

A residues from the rPfSUB1$_{cat}$-ca chain. Calculated with PISA/EBI Bioinformatics.

Supplementary Table 2. rPfSUB1$_{cat}$-ca (+ $CaCl_2$) active site pocket interactions with Prod$_{p9}$. List of hydrogen bonds.

P:ARG 139[NH1]*	3.34	A:ASP 471[OD1]
P:ARG 139[NH2]*	2.97	A:ASP 471[OD1]
P:LYS 176[N]	3.65	A:ASP 477[OD1]
P:ASN 177[N]	3.10	A:ASP 477[OD1]
P:ASN 177[O]	3.61	A:LYS 510[NZ]
P:ASN 179[ND2]	3.61	A:TYR 506[OH]
P9:GLU 209[OE1]	2.60	A:SER 497[OG]
P9:GLU 209[OE2]	2.96	A:GLY 498 [N]
P9:GLU 209[OE2]	3.04	A:ILE 499 [N]
P7:ASP 211[OD1]	2.83	A:GLY 470 [N]
P6:LYS 212[O]	2.85	AS6:LEU 469[N]
P4:VAL 214[N]	2.95	AS4:GLY 467[O]
P4:VAL 214[O]	2.95	AS4:GLY 467[N]
P3:SER 215[N]	3.09	AS3:SER 492[O]
P3:SER 215[O]	2.86	AS3:SER 492[N]
P3:SER 215[O]	3.82	AS3:SER 492[OG]
P3:SER 215[OG]	3.88	AS3:SER 492[O]
P2:ALA 216[N]	3.33	AS2:LYS 465[O]
P1:ASP 217[N]	3.38	AS1:SER490 [O]
P1:ASP 217[O]	3.52	AS1:THR 605[N]
P1:ASP 217[O]	2.59	AS1:ASN 520[ND2]
P1:ASP 217[O]	3.84	AS1:THR 605[OG1]
P1:ASP 217[O]	3.13	AS1:SER 606[N]
P1:ASP 217[OD1]	3.21	AS1:SER 519[OG]
P1:ASP 217[OD2]	2.91	AS1:SER 492[OG]
P1:ASP 217[OD2]	3.08	AS1:SER 517[OG]
P1:ASP 217[OXT]	3.59	AS1:HIS 428[NE2]

* Salt bridges, P residues from the Prod$_{p9}$ chain, P1-P6 Prodomain active site pocket residues, P7 and P9 helix capping residues, A residues from rPfSUB1$_{cat}$-ca chain, AS1-AS6 active site pockets. Calculated with PISA/EBI Bioinformatics.

Supplementary Table 3. Summary of Cys-containing peptides identified by LC-MS/MS analysis of rPfSUB1$_{cat}$ tryptic digests. (**a**) rPfSUB1$_{cat}$ (18.4 µM) was treated for 20 min in the dark at room temperature with iodoacetamide (1 mM) before subjecting the protein to SDS-PAGE under non reducing conditions on a 15% gel. The band corresponding to rPfSUB1$_{cat}$ was excised from the gel, digested with sequencing grade trypsin, and digests analysed by LC-MS/MS. (**b**) rPfSUB1$_{cat}$ (18.4 µM) was treated for 30 min at room temperature with pHMB (2 mM) without prior reduction, before supplementing the reaction with iodoacetamide (1 mM) and proceeding as in (**a**). Note that Cys581 exhibits a strong alkylation signal in both experiments indicating lack of reactivity with pHMB. Cys521 is not alkylated in the second experiment indicating preferential binding to pHMB, although a very small fraction of it is present as not modified.

(a) Control	Peptides (+1 mM Iodoacetamide)	Signal intensity	Modifications
Cys521	GILFFVSASNCSHPK	1.666E6	C11 (Carbamidomethyl)
Cys534	CDLSINAK	1.506E7	C1 (Carbamidomethyl)
Cys581	YCQLAAPGTNIYSTAPHNSYR	2.233E9	C2 (Carbamidomethyl)
(b) pHMB treatment	Peptides (+2 mM pHMB + 1 mM iodoacetamide)	Signal	Modifications
Cys521	GILFFVSASNCSHPK	4.110E5	
Cys534	KCDLSINAK	4.664E5	C2 (Carbamidomethyl)
Cys581	YCQLAAPGTNIYSTAPHNSYR	2.486E8	C2 (Carbamidomethyl)

Supplementary Table 4. List of oligonucleotide primers used for site-directed mutagenesis of PfSUB1. Bases indicated in red are those altered from the wild type sequence in order to encode amino acid mutations.

Primer name	Primer sequence (5′ to 3′)
SUB1-C521A-F	G TCT GCG TCG AAC GCT TCC CAT CCA AAG TCT AG
SUB1-C521A-R	CT AGA CTT TGG ATG GGA AGC GTT CGA CGC AGA C
SUB1-C534A-F	CCA GAT ATC CGC AAG GCT GAC CTG TCT ATC
SUB1-C534A-R	GAT AGA CAG GTC AGC CTT GCG GAT ATC TGG
SUB1-C581A-F	C TCC AAC AAG TAC GCT CAA TTG GCA GGT AC
SUB1-C581A-R	GT ACC TGC CAA TTG AGC GTA CTT GTT GGA G

Supplementary References

1. Withers-Martinez, C. et al. Plasmodium subtilisin-like protease 1 (SUB1): insights into the active-site structure, specificity and function of a pan-malaria drug target. *Int J Parasitol* **42**, 597-612 (2012).
